# Vomiting and wasting disease associated with hemagglutinating encephalomyelitis viruses infection in piglets in jilin, china

**DOI:** 10.1186/1743-422X-8-130

**Published:** 2011-03-21

**Authors:** Wei Gao, Kui Zhao, Chuanbo Zhao, Chongtao Du, Wenzhi Ren, Deguang Song, Huijun Lu, Keyan Chen, Zhiping Li, Yungang Lan, Shengnan Xie, Wenqi He, Feng Gao

**Affiliations:** 1 College of Animal Science and Veterinary Medicine, Jilin University, Changchun 130062, P.R. China; 2 Laboratory Animal Center, Jilin University, Changchun 130062, P.R. China; 3 Key Laboratory of Zoonosis, Ministry of Education, Institute of Zoonosis, Jilin University, Changchun 130062, P.R. China

## Abstract

One coronavirus strain was isolated from brain tissues of ten piglets with evident clinical manifestations of vomiting, diarrhea and dyskinesia in Jilin province in China. Antigenic and genomic characterizations of the virus (isolate PHEV-JLsp09) were based on multiplex PCR and negative staining electron microscopy and sequence analysis of the Hemagglutinin-esterase (HE) gene. These piglets were diagnosed with Porcine hemagglutinating encephalomyelitis virus (PHEV).

Necropsy was performed on the piglets. Major pathological changes included meningeal hyperemia, meningeal hemorrhage and cortical hemorrhage. Minor changes were also observed in other organs. Histopathological changes included satellitosis and neuronophagia in the cerebral cortex.

Mice were infected with the isolated virus. Their histopathological changes were similar to those symptoms observed in the piglets, exhibiting typical changes for non-suppurative encephalitis. Thus, Porcine hemagglutinating encephalomyelitis virus mainly causes damage to the nervous system but also impacts other organs. This viral strain (isolate PHEV-JLsp09) found in the Siping area of Jilin Province in China is evolutionally closest to the HEV-67N stain (North American strain), indicating that this viral strain evolved from the PHEV from North America.

## Background

Porcine hemagglutinating encephalomyelitis coronavirus (PHEV) is a member of the Coronaviridae family, which causes porcine encephalomyelitis. PHEV predominantly affects 1-3 week-old piglets[1], with clinical piglets vomiting, exhaustion and obvious neurological symptoms as the main feature. The mortality rate is up to 20-100%[2]. Since 1958, when the disease broke out in the Canadian province of Ontario for the first time[3], many countries have reported about it. Serological test results proved that it is common for the pigs to be infected by PHEV[4,5], and the disease may have spread worldwide. In August 2006, the disease broke out in part of the pig farms in Argentina, resulting to 1226 deaths, with the morbidity rate up to 52.6%[1]. In China, an PHEV infection has been reported occurring in a pig farm in Beijing as early as in 1985, followed with reports from Jilin, Liaoning, Shandong, Taiwan, etc. The large-scale epidemics of HEV occurred in Taiwan in 1994 had a fatality rate of almost 100%[6], resulting to serious economic losses. Serological survey conducted by foreign scholars revealed that PHEV infection in pigs is very common, with a worldwide distribution[4,5].

Coronaviruses are usually divided into three groups based on genetic and serological relationship [7]. HEV, togather with murine hepatitis virus (MHV), bovine coronavirus (BCoV), human coronavirus OC43 (HcoV-OC43), rat coronavirus (RCoV), belongs to group 2[8].

In this report, the clinical and neuropathologic feathers of spontaneous PHEV infection had been reported. Microscopically, coronavirus-like particles were detected in the supernatant of the brain samples by electron microscopy. One coronavirus strain (isolate PHEV-JLsp09) was isolated from the piglets. The Hemagglutinin- esterase (HE) gene of PHEV strains was amplified by reverse transcription- polymerase chain reaction (RT-PCR) and sequenced. The homology and phylogenetic analyses were done between the group 2 coronaviruses and influenza C virus strains downloaded from Genbank, based on the sequence of HE gene.

## Methods

### Virus samples

Diseased pigs were obtained from a pig farm in Siping City of Jilin Province in China. Four sows farrowed thirty-eight piglets. Among these piglets, 28 piglets produced by 3 sows died. 10 surviving piglets that were produced by one sow were delivered to the laboratory for examination. The 10 Tu San Yuan piglets were all 20 days old and had not been immunized with any vaccines. Clinical signs were vomiting, diarrhea, convulsions and obvious neurological signs. They died within three days of submission. The brain tissues of diseased pigs were collected under sterile conditions and homogenized before adding 1 mL of 0.01 mol/L PBS. The samples were centrifuged at 4°C at 5,000 × g for 30 min. The supernatants were then inoculated into PK-15 cells (ATCC CCL-33) for culture.

### Negative staining electron microscopy

Brain tissues from the diseased pigs were homogenized before adding 1 mL of 0.01 mol/L PBS. The mixtures were centrifuged at 4°C at 5,000 × g for 30 min. The supernatants were collected and adjusted to a total volume of 1 mL with PBS. The samples were then centrifuged at 4°C at 10,000 × g for 15 min. The supernatants were applied to a copper mesh and stained with 2% phosphotungstic acid for 1-2 min before they were examined under an electron microscope.

### Necropsy and histopathological examination

The diseased pigs were dissected and pathological examination was used to assess the lesions. Sectioning of tissues for histopathological examination. Brain, different segments of spinal cord, heart, lung, liver, kidney and other tissues were used for paraffin-embedded sectioning and hematoxylin and eosin (H&E) staining procedures.

### Multiplex RT-PCR

Viral RNA was extracted from cell culture isolates using a QIAamp Viral RNA Mini kit (Qiagen, Valencia, CA). RT-PCR was performed under standard conditions using virus-specific primers. Based on the clinical signs of the diseased pigs, classical swine fever virus (CSFV), porcine hemagglutinating encephalomyelitis virus (PHEV), porcine reproductive and respiratory syndrome virus (PRRSV) and pseudorabies virus (PRV) were selected for further analysis by multiplex PCR. The program used for multiplex PCR was: 95°C denaturation for 5 min; 30 cycles of 94°C denaturation for 50 s, 53°C annealing for 50 s and 72°C extension for 60 s; 72°C extension for 10 min and then storage at 4°C.

Primer sequences are as follows:

CSFV (HCV) primers:

P1: 5'- CCATGCCCATAGTAGGACTAGC -3';

P2: 5'- TGTGATTTCACCCTAGCGACC-3'.

PHEV primers:

P1: 5'- tgaaaatgtttttgcttcct-3'

P2: 5'- ttaagcataatgctgccta-3'

PRRSV primers:

P1: 5' - CCAACAAAACCAGTCCAGAG - 3'

P2: 5' - GTGCCATTCACCACACATTC - 3'

PRV primers:

P1: 5'- CGACAACGAGCTCCTCATCT-3'

P2: 5'-TTCTTCTTGCGCGCCTTGTG - 3'

### Phylogenetic analysis

Comparisons of the field isolate sequences with those of PHEV available in the Genbank database were performed using the online BLAST. The resulting sequences were aligned by ClustalW http://www.ebi.ac.uk/Tools/clustalw/index.html. Deduced amino acid sequences were assembled into a multiple sequence alignment. A phylogenetic tree derived from deduced amino acid sequences was constructed for the PHEV using the bootstrapped maximum parsimony method of MEGA version 4.0 [9].

### Infection of mice with isolated viral strain

Mice were inoculated with isolated viral strain from PK-15 cell culture. Ten mice were randomly divided into five groups of two mice each; one group was set as the control group. The experiment groups received intracerebral inoculations of the virus solution. The dosage of inoculation for each mouse was 2.5 log_10 _TCID_50_/0.1 mL (The TCID_50 _was 10^-3.4^/0.1 mL). The control group was inoculated with 0.1 mL PBS. The clinical signs of the mice were monitored.

### The pathomorphology observation of infected mice

Necropsy of mice was performed, and the pathology of each organ was examined. Brain, spinal cord, lung, liver, spleen and other organs from diseased mice were fixed with 10% neutral formalin and embedded in paraffin for sectioning, H&E staining and microscopic examination.

## Case presentation

### Clinical signs of piglets

The diseased pig cases came from a pig farm in Siping, China. The Tu San Yuan pigs were naturally propagated and raised. The sows mainly showed elevated body temperature and loss of appetite. Their early clinical manifestations were sneezing, coughing, regurgitating, vomiting and spitting up milk. Sick piglets often stayed together, had arched backs and did not like to walk. During late stages of the disease, they suffered from physical deterioration. Piglets with poor resistance would suffer from severe dehydration. Exhibiting difficulty in breathing and cyanosis, they would sink into comas and die. Piglets with better resistance would lose their appetite and soon became emaciated and died. In the current study, 28 piglets died within 20 days of birth. The only 10 surviving piglets were sent to the animal facility of our lab to be raised separately. Their clinical signs were significant increase of body temperature, lethargy, crowding together, eyes half-closed or lying on the ground showing sleepiness and loss of appetite. Nervous system signs of the diseased pigs included nervous whole body muscle convulsions, taking a dog-sitting position and weakness/fainting. During the late stage, diseased pigs wobbled and could not stand, their noses and feet were cyanotic and they discharged watery feces. Some of them lost their eyesight or had opisthotonos and nystagmus. Finally, they experienced breathing problems, lay on their sides exhausted and vomited. The only 10 surviving pigs sent to our lab for examination died within 3 days; the death rate of the piglets was 100%.

### Morphological features

**Figure 1:**
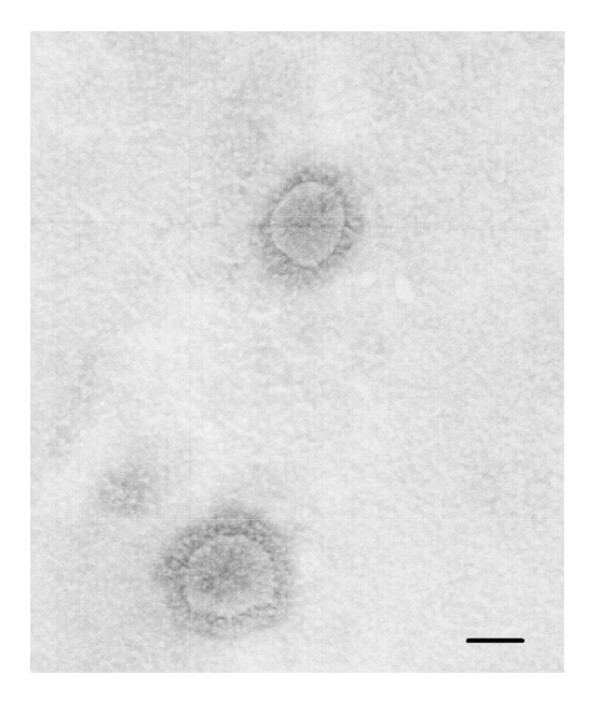
**Typical coronavirus-like viral particles were observed by electron microscopy**. When negatively stained cell culture supernatant was examined under an electron microscope, typical coronavirus-like viral particles with a diameter of about 110-130 nm were observed (bar = 100 nm); these viral-like particles shared similar morphology and size with coronavirus.

Negatively stained cell culture supernatant was examined under an electron microscope, and typical coronavirus-like viral particles with a diameter of about 110-130 nm were observed (Figure 1).

### Macroscopic lesions

**Figure 2:**
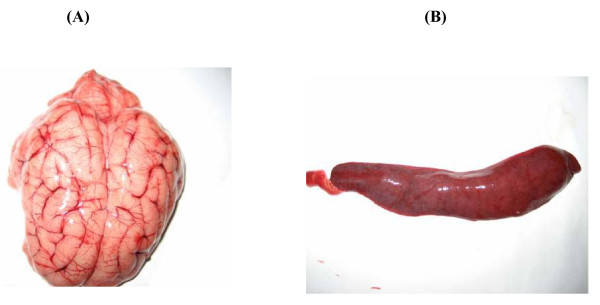
**Macroscopic lesions**. (A) Among the gross defects, all diseased pigs showed meningeal hyperemia, cortical hyperemia and cortical venous congestion. Brain sections showed many dispersed small hematomas, where these were more evident in white matter than in grey matter. (B) The spleen was slightly enlarged.

Grossly visible, all diseased pigs showed meningeal hyperemia, cortical hyperemia and cortical venous congestion (Figure 2A). Brain sections showed many dispersed small hematomas, and these were more evident in white matter than in grey matter. The spleen was slightly enlarged (Figure 2B). There were no significant changes in other organs.

### Microscopic lesions

**Figure 3:**
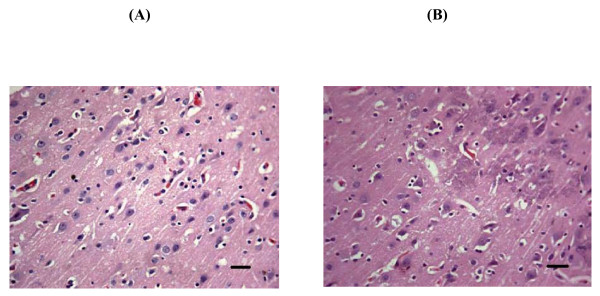
**Microscopic lesions**. (A) Histopathological examination of diseased pigs showed cortical neuronal edema and increased peripheral space (H&E, 400×; bar = 100 μm). (B) Small glial cells surrounded neurons and exhibited neuronophagia and satellitosis (H&E, 400×; bar = 100 μm).

Cortical blood vessels were slightly dilated, there was neuronal edema in the pyramidal and the polymorphic cell layers and the peripheral space increased (Figure 3A). Cell bodies were swollen, and the Nissl bodies were partially dissolved. The neuronal nuclei were concentrated and stained heavily. Mild glial cell proliferation was seen in the small pyramidal cell layer, and some small glial cells surrounded neurons, showing neuronophagia and satellitosis (Figure 3B).

### Multiplex RT-PCR assay

Based on the diseased pigs' clinical signs, multiplex RT-PCR was used for the examination. A 1275-bp positive fragment was identified. This fragment was inserted into cloning vector PMD-18T and sent to the invitrogen company for sequencing. Sequencing results showed that the insert was 99% homologous to the HEV 67N strain nucleotide sequence (Genbank accession number: AY078417.1), and it contained a T to G replacement at position 1172 and a T to C replacement at position 1271, indicating that the piglets were infected by a infection of HEV.

### Phylogenetic analysis

**Figure 4:**
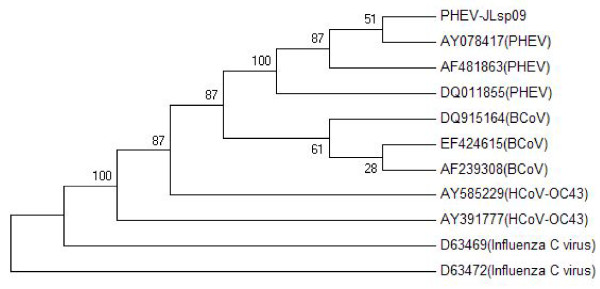
**Phylogenetic trees of hemagglutinating encephamylitis virus HE sequences**. Phylogenetic trees of PHEV based on the complete open reading frames. In trees, the results after bootstrap replicates are indicated, Porcine hemagglutinating encephalomyelitis virus (PHEV-JLsp09/AY07841/AF481863/DQ011855); Bovine coronavirus (DQ915164/EF424615/AF239308); Human coronavirus OC43 (AY585229/AY391777); Influenza C virus (D63469/D63472).

The Hemagglutinin-esterase (HE) gene of the isolate PHEV-JLsp09 strain was sequenced. Sequence alignment with the HEV strains, the representative strains from the group 2 coronaviruses and influenza C viruses from Genbank showed that this PHEV-JL09 strain is evolutionally closest to the HEV-67N strain (North American strain), followed by the HEV IAF-404 strain (Canadian strain), and then the HEV (European strain). Furthermore, this strain is closely related to BCoV and HCoV-OC43 from the group 2 coronaviruses. Influenza C virus is least similar to this virus (Figure 4). Phylogenetic analysis showed that PHEV-JLsp09 strain may originate from the HEV-67N strain.

### Clinical signs of infected mice

After inoculation of the viral solutions, all of the mice started to show distinct nervous signs within 48 h, and they died within 7-9 days of inoculation. The major clinical manifestations included lethargy, loss of appetite, taking a dog-sitting position, hair-bristling, arched back, grasping the mouth or the head with the forelimbs, waving the forelimbs up and down and paralyzed or palsied hind limbs in some mice. Mice in the control group did not show any clinical signs.

### Histopathological examination of infected mice

**Figure 5:**
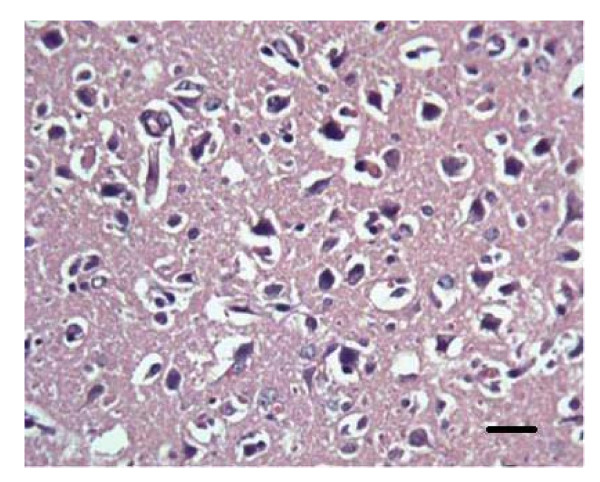
**Histopathological examination of infected mice showed typical changes for non-suppurative encephalitis**. Cerebral cortical neurons were swollen (H&E, 400×; bar = 100 μm).

Histopathological examination showed typical changes for non-suppurative encephalitis. The cerebral cortical neurons were swollen (Figure 5). Neurons were degenerated and necrotic, and there were many proliferated glial cells, eliciting neuronophagia.

## Discussion

Results from the sequence alignment are consistent with the evolution process of the HE gene. The HE gene of coronavirus initially evolved from the HEF gene of influenza C virus[10]. During this process, the HE was transformed from a trimer into a dimer but maintained its acetylcholine esterase domain[10]. Based on the phylogenetic analysis of genes and antigens, bovine coronavirus (BCoV) from the group 2 coronaviruses, human coronavirus (HCoV-OC43) and porcine hemagglutinating encephalomyelitis virus (PHEV) are derived from a common ancestor [8]. Their HE genes continue to evolve in the three different hosts.

PHEV independently evolved in North American and European regions. The viral strain found in the Siping area of Jilin Province in China is evolutionally closest to the HEV-67N stain (North American strain), indicating that this viral strain evolved from the PHEV from North America. In 1984, in a pig farm on the outskirts of Beijing, an HEV infection in the hybrid progenies of imported Landrace, Duroc, and Hampshire pigs was the first case reported in mainland China[11]. In 1985, an HEV infection in the progenies of American Duroc and Hampshire lean pigs occurred at a pig farm in Jilin Province. The epidemiological and clinical characteristics reported in that case were very similar to the HEV cases reported in Canada and United States in the 1980 s [12]. Therefore, we speculate that the viral strain isolated may be a mutated strain of HEV-67N, which was brought by the imported pigs and then spread and mutated in native pigs.

The replication of vomiting and wasting type encephalomyelitis virus initially occurs in nasal mucosa, tonsil, lung, and in some cases, in small intestine. From these invasive sites, the virus reaches the medulla oblongata through the peripheral nervous system, subsequently extends to the entire brain stem and finally reaches the cerebrum and cerebellum. Vomiting is caused by viral replication in the vagal sensory ganglia, whereas wasting occurs because of the vomiting and the delayed stomach emptying. The porcine vomiting and wasting type hemagglutinating encephalomyelitis damages the pig nervous system, as indicated by non-suppurative encephalitis and edema. The pathological changes appear as neuron degeneration, nuclear dissolution and perivascular edema; there are also some instances of satellitosis and neuronophagia.

The mice were inoculated through intracerebral injection using isolated virus, and all of the mice that were infected shared similar clinical signs observed from clinical cases. The major lesions of the infected mice were in the central nervous system; these lesions exhibited typical changes for non-suppurative encephalitis. The cortical neurons were swollen. Neurons were degenerated and necrotic, there was proliferation of glial cells, and typical neuronophagia could be seen.

This outbreak of porcine hemagglutinating encephalomyelitis in a pig farm follows a specific pattern, namely that the disease occurred in the early and middle birth period of first litter gilts; some piglets born during the middle and late birth period did not have the disease. Based on reports from other countries [13], these later piglets may be healthy because sows in the middle and late birth period have acquired an immune response due to earlier infection, and their piglets have obtained passive immunization and are protected through colostrum. Based on this theory, "sub-infection" may prevent the disease. In the "sub-infection" method, at least one month before giving birth, the sows have contact with diseased pigs or attenuated virus by spray or muscular injection to immunize the sows; then, the young piglets can be protected through the antibodies in the colostrum. During the birth period, once the piglets have the disease, they must be separated and treated, and the diseased must be treated.

## Conclusion

From the results of clinical signs, pathological changes and laboratory diagnosis, this outbreak of piglet vomiting and wasting disease in a Siping City, Jilin province, pig farm was caused by hemagglutinating encephalomyelitis virus. With multiplex RT-PCR identification and sequence alignment, we found that this viral strain shares 99% nucleotide homology with a PHEV strain (GenBank accession number: AY078417). In addition, with electron microscopy, we detected coronavirus particles in the brain tissues of both the diseased piglets and mice that were inoculated with the isolated virus. The passaged virus still caused death in the mice, suggesting this pathogen is virulent. Inoculated laboratory mice showed signs similar to those of naturally diseased pigs and to the pathlogical changes of non-suppurative encephalomyelitis.

## Competing interests

The authors declare that they have no competing interests.

## Authors' contributions

WG and KZ carried out most of the experiments and wrote the manuscript. WR, DS, KC, YL and SX participated in the planning of the project. CZ and CD collected the samples. ZL and HL, performed the PCR and phylogenetic analysis. FG and WH conceived of the study and participated in its design and coordination. All authors read and approved the final manuscript.
